# HOW DOES DIGITAL USE AFFECT THE DAILY ACTIVITIES AND LONELINESS AMONG CHINESE OLDER ADULTS?

**DOI:** 10.1093/geroni/igae098.3232

**Published:** 2024-12-31

**Authors:** Xiayu Chen, Zhan Yu, Qingwen Xu

**Affiliations:** University of Illinois Urbana-Champaign, Champaign, Illinois, United States; East China Normal University, Shanghai, Shanghai, China (People’s Republic); New York University, New York, New York, United States

## Abstract

The increasing integration of digital technology into everyday life has significant implications for the aging population. This study explores the relationship between Internet use and the psychosocial well-being of older adults, particularly examines whether digital engagement would have replaced (or enhanced) traditional activities and social interactions, and to what extent digital engagement has contributed to alleviating loneliness among older adults within the Chinese community-dwelling context. Utilizing data from the Chinese General Social Survey, which included 4,283 respondents aged 60 and over, we performed various regression models to examine the relationship among Internet use and daily activities, as well as Internet use and loneliness. Our research reveals the digital divide within the older population in China, where Internet users were the majority those with access to resources and learning and physical capacity, suggesting a deeper concern for technology equity. Our findings also indicate that Internet use among older adults is associated with increased participation in traditional activities, such as shopping, information seeking, and socializing with friends and relatives. Additionally, a higher intensity of Internet use correlates with lower levels of reported loneliness, indicating that Internet use could enhance social connections and overall well-being among older adults. These results highlight the potential of digital technologies to support active aging by fostering enhanced social interactions and engagement in daily life. It calls for developing holistic digital integration strategies and creating an age-friendly digital environment. The findings also provide valuable insights for policymakers and stakeholders in China and potentially in other contexts.

